# Monkeypox: the consequences of neglect

**DOI:** 10.2471/BLT.22.021022

**Published:** 2022-10-01

## Abstract

The monkeypox epidemic is highlighting the risks entailed in dismissing an emerging disease as someone else’s problem. Vijay Shankar Balakrishnan and Gary Humphreys report.

For Dr Dimie Ogoina, the outbreak of monkeypox disease affecting over 100 countries worldwide elicits powerful feelings of déjà vu.

In 2017, working as a clinician in Niger Delta University Teaching Hospital, Bayelsa State, Nigeria, Ogoina diagnosed the index case of Nigeria’s biggest outbreak of the disease.

“An 11-year-old boy came into the hospital in September,” he recalls. “He was covered in lesions. They were on his trunk, face, palms and soles of the feet, as well as in his mouth and nose.”

Dismissing a diagnosis of chickenpox because of a prior infection with the disease, in the days following, Ogoina began to suspect that the boy might be suffering from monkeypox.

Caused by the monkeypox virus, a genus of *Orthopoxvirus* in the Poxviridae family of viruses that includes smallpox, monkeypox has long been characterized as a zoonotic disease transmitted by monkeys or rodents to people living in tropical rainforest areas of central and west Africa.

“The boy and two of his siblings reported handling a neighbor’s monkey one month earlier,” Ogoina recalls. When laboratory samples came back confirming the diagnosis as West Africa clade human monkeypox (now referred to as clade II, one of two clades identified), the case seemed to be conforming to historical precedent.

And then the other cases started coming in.

“Over the next few months there were a total of 228 cases across the country, the sub-population affected being mostly young adults, most of them males, living in urban and semi-urban settings and most of them with no known exposure to animals,” Ogoina says.

Because it seemed unlikely that contact with animals was driving the outbreak, Ogoina and his co-researchers investigated further, discovering that many of the male patients reported engaging in sexual activity with multiple partners, including sex workers, in the period prior to infection.

The researchers raised questions regarding the possibility of sexual transmission of the virus articulated in a report published in the April 2019 issue of PLOS One.

The epidemiological implications of those questions were considerable. Ogoina, now professor of medicine and infectious diseases at Niger Delta University in Nigeria, explains: “Rather than being a pathogen that occasionally jumped from an animal vector into a human and then typically petered out, we were suggesting that monkeypox virus could become a widely spread, sexually transmitted infection.”

Five years later, monkeypox virus has spread.

“The way to address these diseases is to do the work where they are endemic.”

Daniel Bausch

Following nine documented cases of monkeypox exported to Israel, Singapore, the United Kingdom of Great Britain and Northern Ireland, and the United States of America (USA) between 2018 and 2021, a global outbreak began in May 2022.

According to the fifth external situation report, published by the World Health Organization (WHO) on 7 September, as of 4 September, 102 countries, territories and areas in all six WHO regions had reported a total of 52 996 laboratory-confirmed cases, 52 309 of which were in the WHO European Region and the Region of the Americas. Some 18 people were reported to have died of the disease.

How the virus is transmitted from person to person remains unclear.

Studies are underway regarding different possibilities, including transmission through bodily fluids such as semen, breastmilk or blood. “What we do know is that one of the main modes of exposure is through direct contact, close contact, skin-to-skin contact, including sexual contact and face-to-face contact, and exposure to respiratory droplets due to lesions in the mouth,” says Dr Rosamund Lewis, the WHO Technical Lead on monkeypox. Lewis notes, however, that the virus can also be transmitted via fomites such as clothing, bedding and the surfaces of furniture.

Notwithstanding some regional variation, the epidemiological evidence regarding population distribution is much clearer.

According to the WHO report cited, just over 98.2% (26 953/27 449) of cases reporting data on gender were males with a median age of 36 years. Of the cases reporting sexual orientation 95.2% (11 923/12 530) defined themselves as gay, bisexual and other men who have sex with men (MSM). This proportion has remained almost unchanged since the beginning of the outbreak.

Logically, the focused nature of the monkeypox epidemic has elicited focused responses. As Dr David Evans, a virologist specializing in the study of poxviruses at the University of Alberta in Canada, puts it: “We need to nip this in the bud and to do that we need to focus on where the buds are.”

Core to those responses are targeted information strategies designed to mitigate the risks of exposure and transmission, often developed in close collaboration with the communities concerned.

WHO’s Epidemic and Pandemic Preparedness and Prevention department, backed by the Department of Global HIV, Hepatitis and Sexually Transmitted Infections Programmes, is working closely with the MSM community to understand how it can support them in prevention.

For Dr Sylvie Briand, department director, such direct engagement is key. “It is important to co-develop messages with affected communities to ensure that the information is tailored to their needs and concerns and empowers them to manage their risks,” she says.

The downside of such intense focus is that it runs the risk of encouraging discrimination and stigmatization. Instances of this have already occurred, recalling the early stages of the human immunodeficiency virus (HIV) pandemic when the acquired immunodeficiency syndrome (AIDS) was often referred to in discriminatory or stigmatizing terms, framing the syndrome as a “gay disease”.

For Lewis, avoiding stigmatization is a moral and practical imperative. “Apart from the obvious unacceptability of discriminating against and vilifying people burdened with a disease, stigmatization runs the risk of discouraging those people from seeking the health services they need,” she says.

Evans points out that stigmatization does not just harm the stigmatized. “The implicit assumption that an outbreak is something happening to ‘others’ can lull the broader population into a false sense of security increasing their risk of infection,” he says.

In many ways, it is this assumption – that the disease is someone else’s problem – that has facilitated the monkeypox virus’s journey out of what might be termed its natural habitat.

“If the world had been listening to the experts on the ground, we would have been in a better position to manage what is going on at this time.”

Ifedayo Adetifa

Dr Anne Rimoin, an epidemiology professor at University of California Los Angeles, in the USA, who is part of the WHO Strategic Advisory Group of Experts (SAGE) Working Group on smallpox and monkeypox vaccines spells it out. “This virus has been spreading in marginalized and vulnerable populations in Africa for decades, and we've done nothing about it because we think it doesn’t concern us,” she says.

For Rimoin, this neglect is not just unethical, it is dangerous. “To get in front of emerging infectious diseases, we are going to have to prioritize dealing with them where they are emerging and first beginning to spread,” she says.

Daniel Bausch, senior director of emerging threats at FIND, the global alliance for diagnostics, takes a similar view. “We need to get beyond the idea that there’s any disease that’s just a problem in some remote area of Africa or Asia,” he says. “The way to address these diseases is to do the work where they are endemic. It is not only the right thing to do in terms of the local populations, it also makes strategic sense because the world is so connected now.”

In regard to the current monkeypox outbreak, an ideal opportunity to “do the work” would have been between 2017 and 2019, when Nigerian doctors were beginning to notice and document the apparent transition of a zoonotic disease into something else. That opportunity was missed and the research of experts such as Dr Dimie Ogoina largely ignored.

The work has now been initiated.

WHO Director-General Tedros Adhanom Ghebreyesus declared the outbreak to be a Public Health Emergency of International Concern on 23 July 2022. Member States have activated clinical and public health incident responses that include comprehensive case finding, contact tracing, laboratory investigation, isolation and clinical management, vaccination and infection prevention and control measures.

WHO is supporting international coordination and information sharing with Member States and partners and has issued guidance on all these aspects of the response.

Would this work have been necessary if more attention had been paid to the cases that occurred in Nigeria in 2017 or to the research published in 2019?

Dr Ifedayo Adetifa, Director-General of the Nigeria Centre for Disease Control, has his doubts. “Expertise and resources might be disproportionately distributed, but you cannot ignore the experience and the knowledge of colleagues on the ground,” he says. “If the world had been listening to the experts on the ground, we would have been in a better position to manage what is going on at this time.”

**Figure:**
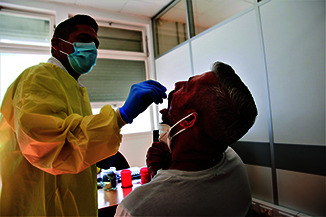
A nurse takes a throat swab from a man who has monkeypox at the Hospital de Santo António dos Capuchos in Lisbon, Portugal.

WHO/Khaled Mostafa

**Figure:**
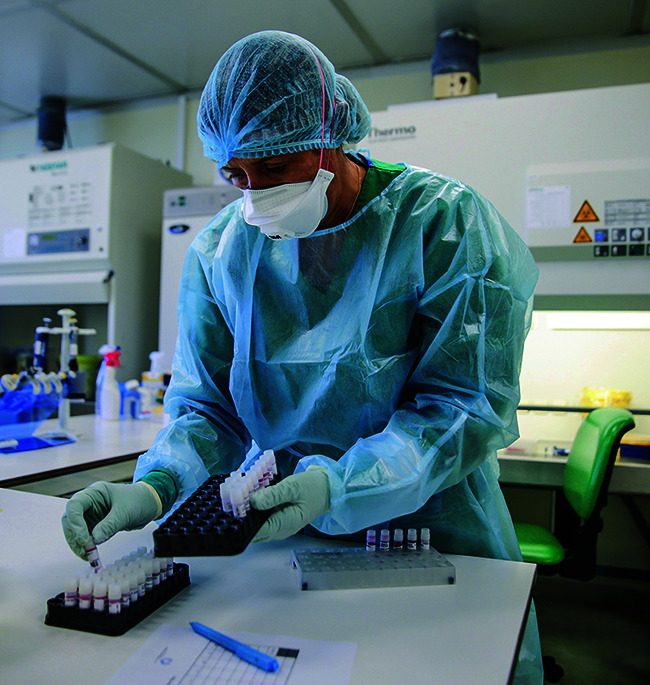
A laboratory technician prepares monkeypox samples for the diagnosis process in the Biosafety Level 3 suite at the National Institute of Health Doctor Ricardo Jorge in Lisbon, Portugal.

WHO/Khaled Mostafa

